# Nest acceptance, clutch, and oviposition traits are promising selection criteria to improve egg production in cage-free system

**DOI:** 10.1371/journal.pone.0251037

**Published:** 2021-05-20

**Authors:** Lorry Becot, Nicolas Bedere, Thierry Burlot, Jenna Coton, Pascale Le Roy

**Affiliations:** 1 NOVOGEN, Plédran, France; 2 PEGASE, INRAE, Institut Agro, Saint Gilles, France; University of Life Sciences in Lublin, POLAND

## Abstract

In cage-free systems, laying hens must lay their eggs in the nests. Selecting layers based on nesting behavior would be a good strategy for improving egg production in these breeding systems. However, little is known about the genetic determinism of nest-related traits. Laying rate in the nests (LRN), clutch number (CN), oviposition traits (OT), and nest acceptance for laying (NAL) of 1,430 Rhode Island Red (RIR) hens and 1,008 White Leghorn (WL) hens were recorded in floor pens provided with individual electronic nests. Heritability and genetic and phenotypic correlations of all traits were estimated over two recording periods–the peak (24–43 weeks of age) and the middle (44–64 weeks of age) of production–by applying the restricted maximum likelihood method to an animal model. The mean oviposition time (MOT) ranged from 2 h 5 min to 3 h and from 3 h 35 min to 3 h 44 min after turning on the lights for RIR and WL hens, respectively. The mean oviposition interval ranged from 24 h 3 min to 24 h 16 min. All heritability and correlation estimates were similar for RIR and WL. Low to moderate heritability coefficients were estimated for LRN (0.04–0.25) and moderate to high heritability coefficients for CN and OT (0.27–0.68). CN and OT were negatively genetically correlated with LRN (-0.92 to -0.39) except during peak production for RIR (-0.30 to +0.43). NAL was weakly to moderately heritable (0.13–0.26). Genetic correlations between NAL and other traits were low to moderate (-0.41 to +0.44). In conclusion, CN and OT are promising selection criteria to improve egg production in cage-free systems. NAL can be also used to reduce the number of eggs laid off-nest in these breeding systems. However, variability in MOT must be maintained to limit competition for the nests.

## Introduction

To provide animals with living conditions that consider their sensitivities better [1], cage-free farming systems were developed for laying hens (barn, free-range, and organic). In the European Union, 50% of laying hens were raised in cage-free systems in 2018 vs. 30% in 2009 and 8% in 1996 [2]. These housing systems provide special breeding conditions, such as social interactions in a large group, but also require the hens to lay in the nests so the eggs can be collected. The laying rate in the nests must be high to guarantee economic security and food safety. For selection purposes, breeders historically raised hens in individual cages, which made it possible to record individual production traits such as egg number and egg quality [3]. Selecting hens for cage-free systems has led to the development of electronic nests that use radio-frequency identification of hens. Electronic nests record, at the individual scale, daily laying rate in the nests as well as “new traits”, such as oviposition (i.e. egg-laying) time, and nesting behavior, such as nest acceptance for laying by hens raised in groups [4–6].

Laying rate, which equals the number of eggs laid divided by the number of days of the recording period, is the trait that has the highest economic weight in breeding programs for laying hens. Laying rate is associated with a clutch, which is defined as consecutive days of oviposition. Successive clutches are separated by one or more days of pause. The length and number of clutches are controlled by the ovulatory cycle, directly observed by the oviposition cycle, and governed by the circadian rhythm and the internal cycle of follicular growth and maturation [7, 8]. Egg-laying can be characterized by two temporal oviposition traits: oviposition time and oviposition interval (i.e. duration between two consecutive ovipositions in the same clutch). The oviposition interval is ca. 24 h but varies greatly. In a population of Rhode Island White that received a lighting regime of 14 h of light and 10 h of darkness, the mean oviposition interval per hen was 21 h 2 min to 27 h 17 min [9]. Highly productive hens have fewer clutches and shorter oviposition intervals, and lay their eggs earlier in the day than less productive hens [7].

Long-term selection has decreased the genetic variability in the laying rate [10]. Since the clutch number and oviposition traits are more basic biological traits than laying rate, they could be more heritable. Studies performed in individual cages have shown that the clutch number, mean oviposition time, and mean oviposition interval were more heritable than the laying rate and were favorably genetically correlated with the laying rate [7, 9–14]. The laying rate could be improved by selecting for these traits in breeding programs. There is little knowledge about the uniformity of oviposition traits [9], but they could also be used to improve the laying rate. Few studies have been performed of cage-free systems, in which hens may compete for the nests [15, 16]. Low to moderate positive genetic correlations (≤ +0.44) were estimated between the number of eggs recorded in individual cages and with electronic nests in floor pens at the peak of production [16]. These results suggest that the laying rate might experience genotype-by-environment interactions.

The total number of saleable eggs from cage-free systems also depends on the acceptance of the nests for laying. Eggs laid off-nest must be collected by hand and decrease the flock’s production because they are usually downgraded or lost. Nest acceptance for laying is influenced by many factors, such as nest design [17], lighting regime [18], enrichment with perches [19], and age of the flock [20–22]. However, little is known about the genetic determinism of this trait because of the difficulty in recording eggs laid off-nest by individual hens. A study of brown-egg layers raised in floor pens of 18 sisters or sire-half-sisters estimated heritability of 0.39 and 0.44 for the percentage of eggs laid off-nest daily and weekly, respectively [21]. These results are promising for selecting hens against off-nest laying.

Continuous recording of the laying activity of hens by electronic nests allows these “new” egg-production traits to be observed. The present study aimed to identify the most relevant traits for improving egg production in cage-free systems through the estimation of genetic parameters for traits recorded by electronic nests in a cage-free environment. To represent a wide phenotypic and genetic variability, populations from two pure lines–Rhode Island Red (RIR) and White Leghorn (WL)–were studied, as were two breeding periods: peak production (24–43 weeks of age) and the middle of production, when the laying rate starts to decrease (44–64 weeks of age).

## Materials and methods

### Ethics statement

All data were registered as part of the commercial and selection activities of Novogen (Plédran, France). These animals and the scientific investigations described herein are therefore not to be considered as experimental animals per se, as defined in European Union directive 2010/63 and subsequent national application texts. Consequently, we did not seek ethical review and approval of this study as regarding the use of experimental animals. All animals were reared in compliance with national regulations pertaining to livestock production and according to procedures approved by the French Veterinary Services.

### Birds

RIR (brown-egg layers) and WL (white-egg layers) are the two main breeds of laying hens used for egg production in the world. This study involved two pure lines–a RIR line and a WL line–created and selected by the Novogen breeding company (Plédran, France). These two lines are specifically selected for egg number, egg quality, body weight, and behavior-related traits such as feather pecking. Data were obtained from 2017–2019 for three batches of RIR, which contained 1,430 laying hens (146 sires and 546 dams), and two batches of WL, which contained 1,008 laying hens (100 sires and 359 dams; Table 1). The known pedigree used to estimate genetic parameters included seven (3,433 individuals) and five (2,354 individuals) generations for RIR and WL, respectively. Hens were raised on the nucleus farm in a barn with floor pens (one pen per line) from 17–64 weeks of age. The mean body weight at 17 weeks of age was 1,415 g and 1,120 g for RIR and WL hens respectively. In the floor pens, hens were raised with cocks (i.e. ca. one cock for ten hens) because the birds were breeders, and eggs were collected for artificial incubation. The lighting regime was the standard 16 h of light and 8 h of darkness. The ambient temperature in the barn was maintained to 20°C. Hens were fed *ad libitum* with a commercial feed (11.47 MJ/kg metablolizable energy, 16.5% of crude protein, 3.70% of Ca).

**Table 1:** Data recorded by individual electronic nests.

	Rhode Island Red			White Leghorn	
Batch	A	B	C	D	E
Number of hens	477	421	532	489	519
Age (wks.)	44–64	24–58	24–64	24–64	24–64
Data recorded*	39,696	73,404	118,665	89,628	122,597
Density (hens/nest)	5.15	4.55	4.60	6.41	4.98

*Correspond to nest visits with oviposition, which were used to define and calculate traits.

### Data recording system

Floor pens of the barn were equipped on two levels (top and bottom) with individual electronic nests (20 Width × 40 Length × 27 Height cm). The density was about 5 hens for 1 nest (Table 1). These nests were developed and optimized by Novogen to record individual nesting behavior. The data recording system used radio-frequency identification with a specific antenna for each nest and a specific transponder for each hen tagged on one leg. A hen’s transponder was automatically recorded when the hen visited a nest. For each visit, the system recorded the hen’s identification, the time of entry in the nest, and the time of exit in the nest. An egg collection system connected to each nest recorded nest visits, with oviposition or not, and the oviposition time. Egg number per nest was recorded manually each day to double-check the number of oviposition times recorded. Data were recorded from 44–64 weeks of age for batch A and 24–64 weeks of age for batches B, C, D, and E (Table 1).

In the present study, only the time of entry for nest visits with oviposition was used to define and calculate traits, for two reasons. First, oviposition time may be incorrect if the egg laid remains stuck in the nest until or after the hen leaves the nest. When this occurs, the oviposition time recorded by the egg collection system is later than the true oviposition time. These data had to be excluded for analyses, unlike the corresponding time of entry in the nest. Second, when the oviposition time was known to be correct, the time of entry in the nest correlated strongly with oviposition time for both lines (phenotypic correlation = +0.99). To facilitate comparison of mean oviposition time (MOT) with results in the literature, we calculated phenotypes of MOT in the nests when oviposition time was known to be correct (see results in Table 1), which represented 90.2% and 87.4% of the data recorded for the RIR and WL lines, respectively.

### Traits

Data were divided into two breeding periods of ca. 140 days: peak egg production (24–43 weeks of age, period 1) and the middle of egg production (44–64 weeks of age, period 2). For all traits, only data for hens that survived for at least half of a given period were kept for analysis; consequently, 0.5–1.1% of the data for each period and each line were excluded. Moreover, an infection in batch B from 59–64 weeks of age strongly influenced laying rate and nesting behavior; thus, data from these six weeks were excluded. The total amount of data recorded (after quality control) varied by batch (Table 1).

The first trait studied was the laying rate in the nests (LRN). For each hen, LRN was calculated by dividing the number of eggs laid in the nests by the number of days alive during a given period and multiplying the result by 100. LRN was analyzed only for hens with LRN ≥ 50% to exclude those that laid in the nests less frequently. Several phenomena can explain why hens do not lay in the nests, such as a proclivity to lay off-nest, molting, or repeated pauses. It was difficult to differentiate these phenomena for hens with low LRN, and they could increase uncertainty in interpretation of LRN. Thus, LRN was used to evaluate the “good” layers in the nests. The second trait studied was the clutch number (CN), which required a preliminary quality control to define it (S1 Appendix). CN was calculated for hens with LRN ≥ 50%.

To characterize oviposition, four traits were calculated from the time of entry in the nest, for visits with oviposition: MOT, logarithm of the variance of oviposition time (LVOT), mean oviposition interval (MOI), and logarithm of the variance of oviposition interval (LVOI). The oviposition interval is the duration between two consecutive times of entry in the nests for visits with oviposition (i.e. for two consecutive days). Hens with less than 10 data available to calculate oviposition traits were excluded from analysis; thus, 0.05–0.10% of data for each period and each line were excluded.

Finally, nest acceptance for laying (NAL) was defined to estimate the ability of hens to lay eggs in the nests. Hens with LRN < 50% or ≥ 50% were assigned the value 0 or 1, respectively.

### Models

Distributions of the data were transformed towards a normal distribution using the “bestNormalize” R package [23]. Genetic parameters were estimated using the mixed linear model:

y=Xb+Za+e

where ***y*** is the vector of observations for the traits analyzed; **X** and ***b*** are the incidence matrix and vector, respectively, related to fixed effects of the hen’s hatch date; **Z** and ***a*** are the incidence matrix and vector, respectively, related to random additive genetic effects; *a* ~ N(0,v(*a*)×A), where v(*a*) is the variance of additive genetic effects and A is the kinship matrix; ***e*** is the vector of random residual effects and *e* ~ N(0,v(*e*)×I), where v(e) is the residual variance and I is the identity matrix.

Phenotypic correlations between traits were calculated using the formula [24]:

$$r_{p}=h_{i}h_{j}r_{a}+e_{i}e_{j}r_{e}$$

where **r**_**p**_, **r**_**a**_, and **r**_**e**_ are phenotypic, additive genetic, and residual correlations, respectively, between traits i and j; **h** is the square root of the heritability for traits i and j; **e** is the square root of the proportion of residual variance for traits i and j.

Genetic parameters were estimated by applying the restricted maximum likelihood method (REML) to an animal model. For this, the tools REMLF90 (EM-REML algorithm) and AIREMLF90 (AI-REML algorithm) of the BLUPF90 family of programs [25] have been used. First, the variance and covariance components were estimated by bivariate animal models using REMLF90. Then, the estimated values were used to calculate phenotypic correlations. Finally, standard errors of heritability and genetic correlations were estimated with AIREMLF90, using the endpoint of REMLF90 as a starting point. GIBBS1F90 [25] was used to estimate a few standard errors (see results in Table 3a) that could not be estimated with AIREMLF90, but it was not used to estimate the other standard errors because it runs slower than AIREMLF90.

## Results

### Phenotypic distributions of nest-related traits

Phenotypic distributions of LRN, CN, MOT, LVOT, MOI, LVOI, NAL were calculated for both lines and breeding periods (Table 2). Mean LRN ranged from 85.90 to 92.33 depending on the line and the breeding period. Mean CN ranged from 2.00 to 3.10 and 3.92 to 5.42 for the RIR and WL lines, respectively. In both lines, a maximum of 23 clutches was reached in the middle of production. MOT ranged from 2 h 5 min to 3 h and 3 h 35 min to 3 h 44 min after turning on the lights for the RIR and WL lines, respectively. However, four RIR hens laid their eggs in the nests 1–7 min before the lights were turned on during peak production, which resulted in some negative MOT values (Table 2). These hens entered the nests an average of 14–20 min before the lights were turned on (S1 Table). MOI was close to 24 h for the RIR and WL lines (24 h 3 min to 24 h 16 min, respectively). NAL exceeded 86%, except in the middle of production for the RIR line (78%).

**Table 2:** Phenotypic data statistics for the Rhode Island Red and White Leghorn lines.

	Rhode Island Red					White Leghorn				
Trait	n^a^	Mean	SD^b^	Min^c^	Max^d^	n^a^	Mean	SD^b^	Min^c^	Max^d^
LRN1^e^ (%)	821	92.33	7.77	50.00	100.00	884	88.97	8.53	50.00	99.00
LRN2^e^ (%)	1,055	92.31	7.65	51.45	100.00	842	85.90	9.56	50.00	100.00
CN1^e^	821	2.00	1.73	1.00	19.00	884	3.92	2.81	1.00	22.00
CN2^e^	1,055	3.10	2.81	1.00	23.00	842	5.42	3.82	1.00	23.00
MOT1^e^ (hh:mm)	848	02:05	01:11	-00:07^f^	05:26	928	03:44	01:15	00:28	07:10
MOT2^e^ (hh:mm)	1,121	03:00	01:25	00:11	06:46	886	03:35	00:59	00:27	06:29
LVOT1^e^ (sq. h^g^)	848	0.05	0.95	-4.57	3.37	928	0.64	0.74	-1.70	3.04
LVOT2^e^ (sq. h^g^)	1,121	0.23	0.95	-3.75	3.06	886	0.73	0.74	-2.18	3.27
MOI1^e^ (hh:mm)	842	24:03	00:08	23:32	25:32	922	24:11	00:14	23:56	26:22
MOI2^e^ (hh:mm)	1,101	24:08	00:14	23:40	26:17	877	24:16	00:19	23:42	26:07
LVOI1^e^ (sq. h^e^)	842	-1.66	0.64	-4.04	0.24	922	-0.79	0.50	-2.43	1.07
LVOI2^e^ (sq. h^e^)	1,101	-1.40	0.71	-3.71	0.53	877	-0.75	0.50	-3.25	0.74
	n^a^	0	1			n^a^	0	1		
NAL1^e^ (%)	953	14	86			1 008	12	88		
NAL2^e^ (%)	1,361	22	78			944	11	89		

^a^Number of hens per trait

^b^Standard deviation

^c^Minimum value

^d^Maximum value

^e^LRN, laying rate in the nests; CN, clutch number; MOT, mean oviposition time; LVOT, logarithm of the variance of oviposition time; MOI, mean oviposition interval; LVOI, logarithm of the variance of oviposition interval; NAL, nest acceptance for laying (0 for hens with LRN < 50%, 1 otherwise). The number after abbreviations indicates the breeding period: 1 for the peak (24–43 wks. of age) and 2 for the middle (44–64 wks. of age) of production.

^f^MOT before the lights were turned on.

^g^Squared hours

### Laying patterns types in the nests

**Fig 1:**
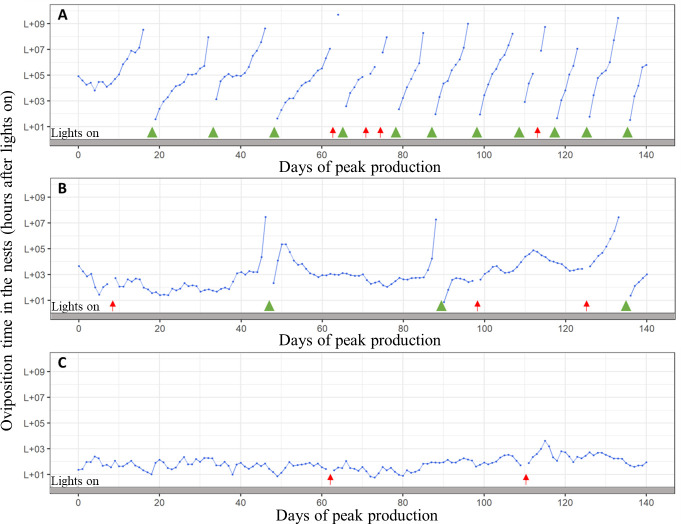
Laying patterns in the nests of three hens (A: short clutches, B: long clutches, and C: one long clutch without pause days) recorded with individual electronic nests for the 140 days of peak production (24–43 weeks of age). Blue points represent eggs laid, and blue lines connect eggs when there is no day without data that separate the eggs (i.e. a pause day (green triangles) or an egg laid off-nest (red arrows)).

Hens’ laying patterns in the nests varied greatly during the breeding season. We classified them into three types based on the CN: hens with more than four short clutches and many pauses, with two to four long clutches, or with one long clutch without pauses (Fig 1).

At peak production, most RIR hens showed the pattern without pauses (54%) or with a few long clutches (39%; Table 3). In the middle of production, most RIR showed the pattern with a few long clutches (46%) or with one long clutch without pauses (34%). At the peak and in the middle of production, 16% and 9% of WL hens, respectively, showed the pattern without pauses, while 31% and 49%, respectively, showed the pattern with more than four clutches. For the RIR line, the MOT of hens with one long clutch ranged from 7 min before to 5 h 43 min after turning on the lights, which yielded a time slot of 5 h 50 min (S2 Table). In the WL line, the MOT of these hens ranged from 32 min to 5 h 21 min after turning on the lights, which yielded a time slot of 4 h 49 min (S2 Table).

**Table 3:** Percentage of hens from Rhode Island Red and White Leghorn lines that displayed a given laying pattern in the nests at the peak and middle production.

	Rhode Island Red (%)		White Leghorn (%)	
Laying pattern in the nests	peak	middle	peak	middle
A (> 4 clutches)	7	20	31	49
B (2–4 clutches)	39	46	53	42
C (1 clutch)	54	34	16	9

Data correspond to hens with a laying rate in the nests ≥ 50% during the peak or middle production period.

### Heritability estimates for nest-related traits

Most nest-related traits displayed moderate to high heritability estimates in both lines. Heritability estimates for LRN were low in RIR (0.04 or 0.10 for the peak and middle periods, respectively; Table 4) and moderate in WL (0.25 for both periods; Table 5). MOT had the highest heritability (0.49–0.68). Heritability estimates for NAL were low to moderate (0.13–0.26). In RIR, heritability estimates were higher in the middle than at the peak of production for CN and oviposition traits. Similar results were observed in WL, except for MOT and LVOT. In both lines, heritability estimates for LRN and NAL were lower in the middle than at the peak of production.

**Table 4:** Heritability estimates (Mean ± SEM; on diagonal), genetic correlations (Mean ± SEM; above diagonal) and phenotypic correlations (below diagonal) for the nest-related traits at the peak (a) and middle (b) production period in the Rhode Island Red line.

**(a)**	Rhode Island Red—peak production (24–43 wks. of age)						
	LRN1^a^	CN1^a^	MOT1^a^	LVOT1^a^	MOI1^a^	LVOI1^a^	NAL1^a^
LRN1	**0.10 ± 0.06**	-0.28 ± 0.27^b^	0.43 ± 0.26^b^	-0.11 ± 0.29^b^	0.27 ± 0.28^b^	-0.30 ± 0.29^b^	0.22 ± 0.40^b^
CN1	-0.28	**0.43 ± 0.09**	0.66 ± 0.11	0.71 ± 0.12	0.74 ± 0.11	0.83 ± 0.10	0.28 ± 0.16
MOT1	0.05	0.36	**0.62 ± 0.09**	0.74 ± 0.09	0.86 ± 0.09	0.48 ± 0.15	0.13 ± 0.18
LVOT1	-0.22	0.54	0.62	**0.33 ± 0.08**	0.75 ± 0.11	0.78 ± 0.11	-0.10 ± 0.23
MOI1	-0.06	0.50	0.51	0.46	**0.34 ± 0.08**	0.42 ± 0.21	0.38 ± 0.21
LVOI1	-0.26	0.60	0.48	0.67	0.48	**0.27 ± 0.08**	-0.12 ± 0.25
NAL1	NA^c^	NA^c^	-0.07	-0.09	-0.02	-0.13	**0.26 ± 0.07**
**(b)**	Rhode Island Red—middle of production (44–64 wks. of age)						
	LRN2^a^	CN2^a^	MOT2^a^	LVOT2^a^	MOI2^a^	LVOI2^a^	NAL2^a^
LRN2	**0.04 ± 0.06**	-0.92 ± 0.03	-0.60 ± 0.10	-0.82 ± 0.06	-0.87 ± 0.04	-0.84 ± 0.05	-0.29 ± 0.13
CN2	-0.49	**0.46 ± 0.08**	0.73 ± 0.08	0.87 ± 0.06	0.96 ± 0.03	0.91 ± 0.05	0.23 ± 0.14
MOT2	-0.24	0.50	**0.68 ± 0.08**	0.82 ± 0.06	0.76 ± 0.07	0.72 ± 0.08	0.19 ± 0.15
LVOT2	-0.45	0.70	0.64	**0.38 ± 0.07**	0.92 ± 0.04	0.93 ± 0.04	-0.06 ± 0.19
MOI2	-0.42	0.81	0.58	0.75	**0.40 ± 0.07**	0.94 ± 0.04	0.16 ± 0.19
LVOI2	-0.47	0.71	0.55	0.81	0.77	**0.34 ± 0.07**	0.04 ± 0.20
NAL2	NA^c^	NA^c^	-0.07	-0.17	-0.06	-0.08	**0.22 ± 0.05**

^a^LRN, laying rate in the nests; CN, clutch number; MOT, mean oviposition time; LVOT, logarithm of the variance of oviposition time; MOI, mean oviposition interval; LVOI, logarithm of the variance of oviposition interval; NAL, nest acceptance for laying. The number after abbreviations indicates the breeding period: 1 for the peak and 2 for the middle production.

^b^Standard errors estimated with GIBBS1F90 because they could not be estimated with AIREMLF90.

^c^Phenotypic correlations for NAL with LRN and CN could not be estimated because all hens with LRN and CN records had a value of 1 for NAL.

**Table 5:** Heritability estimates (Mean ± SEM; on diagonal), genetic correlations (Mean ± SEM; above diagonal) and phenotypic correlations (below diagonal) for the nest-related traits at the peak (a) and middle (b) of production in the White Leghorn line.

**(a)**	White Leghorn—peak of production (24–43 wks. of age)						
	LRN1^a^	CN1^a^	MOT1^a^	LVOT1^a^	MOI1^a^	LVOI1^a^	NAL1^a^
LRN1	**0.25 ± 0.07**	-0.74 ± 0.15	-0.39 ± 0.15	-0.67 ± 0.13	-0.61 ± 0.15	-0.88 ± 0.15	0.44 ± 0.19
CN1	-0.42	**0.35 ± 0.08**	0.64 ± 0.11	0.89 ± 0.05	0.97 ± 0.02	0.92 ± 0.07	-0.03 ± 0.17
MOT1	-0.34	0.44	**0.58 ± 0.08**	0.70 ± 0.09	0.73 ± 0.09	0.74 ± 0.10	-0.08 ± 0.18
LVOT1	-0.45	0.75	0.51	**0.40 ± 0.08**	0.92 ± 0.04	0.90 ± 0.06	-0.22 ± 0.20
MOI1	-0.41	0.85	0.56	0.77	**0.37 ± 0.08**	0.88 ± 0.07	-0.16 ± 0.21
LVOI1	-0.42	0.67	0.44	0.73	0.71	**0.29 ± 0.08**	-0.33 ± 0.22
NAL1	NA^b^	NA^b^	-0.10	-0.17	-0.12	-0.12	**0.24 ± 0.06**
**(b)**	White Leghorn—middle of production (44–64 wks. of age)						
	LRN2^a^	CN2^a^	MOT2^a^	LVOT2^a^	MOI2^a^	LVOI2^a^	NAL2^a^
LRN2	**0.25 ± 0.07**	-0.85 ± 0.11	-0.55 ± 0.16	-0.78 ± 0.12	-0.75 ± 0.14	-0.79 ± 0.14	0.31 ± 0.36
CN2	-0.54	**0.45 ± 0.09**	0.62 ± 0.11	0.97 ± 0.03	0.94 ± 0.03	0.87 ± 0.07	-0.23 ± 0.29
MOT2	-0.29	0.43	**0.49 ± 0.08**	0.67 ± 0.11	0.74 ± 0.09	0.64 ± 0.12	0.02 ± 0.26
LVOT2	-0.56	0.83	0.43	**0.34 ± 0.08**	0.93 ± 0.04	0.84 ± 0.08	-0.22 ± 0.30
MOI2	-0.51	0.87	0.53	0.76	**0.45 ± 0.08**	0.85 ± 0.07	-0.41 ± 0.36
LVOI2	-0.50	0.69	0.36	0.70	0.67	**0.33 ± 0.08**	-0.37 ± 0.34
NAL2	NA^b^	NA^b^	-0.10	-0.17	-0.12	-0.12	**0.13 ± 0.06**

^a^LRN, laying rate in the nests; CN, clutch number; MOT, mean oviposition time; LVOT, logarithm of the variance of oviposition time; MOI, mean oviposition interval; LVOI, logarithm of the variance of oviposition interval; NAL, nest acceptance for laying. The number after abbreviations indicates the breeding period: 1 for the peak and 2 for the middle production.

^b^Phenotypic correlations for NAL with LRN and CN could not be estimated because all hens with LRN and CN records had a value of 1 for NAL.

### Relationships between nest-related traits

Strong negative genetic correlations were estimated between LRN and other nest-related traits in both lines and periods. The strongest genetic correlations were estimated for CN (-0.92 to -0.74), LVOT (-0.82 to -0.67), MOI (-0.87 to -0.61), and LVOI (-0.88 to -0.79), except for peak production in RIR (-0.30 to +0.27 between these traits and LRN). CN, LVOT, MOI, and LVOI were strongly positively correlated (+0.42 to +0.97). In both lines, genetic correlations between LRN and CN or oviposition traits were stronger in the middle than at the peak of production, except for LVOI in WL. NAL was weakly to moderately correlated with LRN, CN, and oviposition traits (-0.41 to +0.44). Phenotypic correlations were lower but consistent with genetic correlations. Phenotypic correlations for NAL with LRN and CN could not be estimated because all hens that were kept for analysis of LRN and CN performances had a value of 1 for NAL.

### Relationships between recording periods for the same trait

Most of the nest-related traits displayed a strong genetic correlation between the two periods despite modest phenotypic correlation (Table 6). In both lines, genetic correlations for MOT, MOI, and NAL between the peak and middle production were close to 1.00 (+0.84 to +0.97). Genetic correlations were strong for all other traits (+0.68 to +0.79). Phenotypic correlations were lower than estimated genetic correlations (+0.27 to +0.83).

**Table 6:** Genetic correlations (Mean ± SEM; r_g_) and phenotypic correlations (r_p_) for the nest-related traits recorded at the peak and middle production for Rhode Island Red and White Leghorn lines.

	Rhode Island Red		White Leghorn	
Traits	r_g_	r_p_	r_g_	r_p_
LRN1—LRN2	0.68 ± 0.14	0.27	0.75 ± 0.20	0.34
CN1—CN2	0.75 ± 0.10	0.49	0.93 ± 0.07	0.54
MOT1—MOT2	0.93 ± 0.02	0.83	0.86 ± 0.04	0.78
LVOT1—LVOT2	0.93 ± 0.09	0.47	0.79 ± 0.10	0.45
MOI1—MOI2	0.94 ± 0.08	0.49	0.84 ± 0.08	0.53
LVOI1—LVOI2	0.69 ± 0.14	0.39	0.90 ± 0.11	0.41
NAL1—NAL2	0.96 ± 0.06	0.77	0.97 ± 0.02	0.68

LRN, laying rate in the nests; CN, clutch number; MOT, mean oviposition time; LVOT, logarithm of the variance of oviposition time; MOI, mean oviposition interval; LVOI, logarithm of the variance of oviposition interval; NAL, nest acceptance for laying. The number after abbreviations indicates the breeding period: 1 for the peak and 2 for the middle of production.

## Discussion

This study estimated moderate to high heritability for CN and oviposition traits in a cage-free system, and strong genetic correlations between these traits and LRN. Therefore, nest-related traits hold potential as selection criteria to improve egg production in cage-free system. Moreover, low to moderate heritability estimates of NAL and low to moderate genetic correlations between it and other nest-related traits suggest that selection can improve this trait.

### Individual electronic nests record nest-related traits

In the present study, phenotypic data recorded by individual electronic nests in a cage-free system are consistent with those in the literature. Here, MOT ranged from 2 h 5 min to 3 h 44 min after turning on the lights, which is consistent with times reported in individual cages or other cage-free systems. In individual cages, 51% of RIR eggs are laid 30 min to 3 h 30 min after turning on the lights [26]. Likewise, using electronic nests in a cage-free system, brown-egg layers reached maximum daily egg production 3 h after turning on the lights [16]. However, the white-egg layers reached the maximum daily egg production 6 h after turning on the lights [16], which suggests that this trait has different phenotypes. The MOI observed are also consistent with those in the literature. For instance, MOI measured using electronic nests ranged from 24 h 6 min to 24 h 10 min in three flocks of brown-egg layers and one flock of white-egg layers [15]. Comparing CN phenotypes with those in the literature is more difficult because the period recorded during hens’ lifetimes varies greatly and CN increases with age [10, 11, 27]. For instance, the mean CN recorded in individual cages from puberty to ca. 90 weeks of age was 29.2 and 43.4 for RIR and WL hens, respectively [10].

The three laying patterns in the nests identified in the present study are also consistent with those in the literature [7]. In a previous study, laying patterns in individual cages were characterized for three lines: two WL lines selected for egg number and egg mass, respectively, and a RIR line selected for egg mass and food consumption [7]. After observing 313 WL and 158 RIR, three patterns were identified from 31–51 weeks of age: 2–3 eggs per clutch (5.7% of hens), more than 3 eggs per clutch (85%), and continuous laying without pauses (9.3%, mainly in the line selected for egg number). Similar laying patterns were observed in the present study with electronic nests and more hens. Intensive selection for egg number may explain why up to 54% of hens laid only one clutch at peak production. However, the high percentage (20% in RIR and 49% in WL lines) of hens with more than four clutches in the middle of production suggests potential for improvement that reduces CN and increases clutch length.

### Nest-related traits are moderately to highly heritable

Heritability estimates were moderate to high for nest-related traits and similar for RIR and WL lines, although they are distant genetic types. Moderate heritability estimates for CN have also been reported in the literature for hens in individual cages. Heritability of 0.46 ± 0.02 for CN was estimated in a population of 5,826 dwarf brown-egg layers selected for 16 generations for increased average clutch length [12]. These results were recently confirmed for a large population of 23,809 RIR and 22,210 WL, which had heritability of CN of 0.42 ± 0.02 and 0.41 ± 0.02, respectively [10]. However, lower heritability (0.15) was estimated for CN with electronic nests but with fewer hens (355; [15]). The average and maximal clutch length, both calculated as a number of eggs, were also studied to characterize clutch traits, and their heritability estimates were close to or lower than those of CN [9–12, 14, 15].

Moderate to high heritability estimates for MOT (0.49–0.68) are relatively consistent with the heritability previously estimated for this trait. Heritability values of 0.81 ± 0.20 and 0.72 ± 0.23, respectively in a line selected for egg mass and a line selected for egg mass and food consumption, were estimated when heritability was lower in a line selected for egg number (0.19 ± 0.17; [7]). Moderate heritability of MOI (0.42 ± 0.23 to 0.55 ± 0.24) was found for these three lines, which are similar to heritability estimates in the present study (0.34–0.45). However, lower heritability (≤ 0.13) was estimated for this trait with a lighting regime of 14 h of light and 10 h of darkness [9], and with electronic nests [15]. In addition, heritability estimates of the uniformity of oviposition traits in the present study are similar to those of mean oviposition traits, but lower. For hens in individual cages, heritability estimates of 0.13 ± 0.05 and 0.18 ± 0.02 for MOI and the variance of oviposition interval, respectively, were reported [9].

Finally, heritability estimates for CN and oviposition traits were higher than those for LRN. These results are consistent with heritability estimates in individual cages for CN and the total number of saleable eggs [10], and for MOT, MOI, and egg number [7]. To our knowledge, the NAL trait has not been reported before in the literature. The present study estimated low to moderate heritability for NAL. Heritability estimates similar to those in individual cages confirm the quality of the recording system of electronic nests, and the moderate heritability estimates for CN are indirect evidence of the quality of the thresholds used to differentiate pause days and eggs laid off-nest (S1 Appendix).

### Laying rate and nest-related traits are strongly genetically correlated

Strong negative genetic correlations for LRN with CN and oviposition traits were estimated in this study. This is consistent with results for individual cages, in which moderate to strong negative genetic correlations were estimated between CN and the total number of saleable eggs (-0.55 ± 0.01 for RIR and -0.28 ± 0.01 for WL; [10]), between MOI and initial egg production (-0.37 for Rhode Island White line; [9]), and between CN and laying rate (-0.54 ± 0.05 for dwarf brown-egg layers; [12]). Using electronic nests, genetic correlations of -0.53 and -0.29 between egg number and CN and MOI, respectively, for brown-egg layers were also estimated [15]. In the present study, the lower and positive genetic correlation at peak production in RIR (-0.30 to +0.43) can be explained by the high LRN and the high frequency of laying in the nests without pauses during this period (54%). Similar results were found for Rhode Island White hens raised until 38 weeks of age in individual cages, with estimated genetic correlations between CN and egg number of -0.43 and +0.38 for the 1995/1996 and 1996/1997 generations, respectively [11]. Genetic correlations between CN and average clutch length were close to -1 (-0.99 to -0.83) for hens raised in individual cages [9–12, 14] or cage-free systems [15]. The same genetic correlations were observed between CN and maximal clutch length (-0.87 to -0.81; [9, 10]). Finally, genetic correlations between NAL and the other traits analyzed were low to moderate, suggesting that NAL had a different genetic background.

### The middle of production is the best period to improve nest-related traits

Genetic correlations for nest-related traits between the peak and middle production are strong. This suggests the potential to group peak and middle production into a single period or to use records from only one period to assess breeding values of selection candidates for nest-related traits. The latter option would decrease costs by decreasing the operating time of the recording system and generating fewer data to store. In this study, the middle of production (i.e. 44–64 weeks of age) seems to be the most favorable breeding period for phenotypes (i.e. most of the variability) and genetic parameters of CN and oviposition traits (i.e. higher heritability and stronger genetic correlations with LRN).

### Nest-related traits are promising selection criteria to improve egg production in cage-free systems

Selecting hens as breeders with a low CN, low MOT, short MOI, and low variance in oviposition traits will help improve LRN for hens in cage-free systems. These traits are also strongly and favorably correlated with each other, indicating that there are no negative interactions for selection. We suggest that CN and MOT are the most favorable traits for increasing egg production in cage-free systems, since they tend to have higher heritability and stronger genetic correlation with LRN than other traits.

However, hens may compete for the nests in cage-free systems, which risks increasing off-nest laying and suffocation of hens in the nests. Selecting for CN and oviposition traits, which are strongly genetically correlated with MOT can increase this competition. In this study, hens in the RIR and WL lines that laid in the nests without pauses had long time slot of MOT (5 h 50 min and 4 h 49 min, respectively). It would be important to maintain this long time slot for highly productive hens, which may help maintain the variability in oviposition time and control nest occupancy rates during the day. A study of the variability in oviposition time suggests that the time slot of laying can be increased by divergent selection of MOT and combining different genotypes in the same flock [13]. Secondly, selecting hens that lay their eggs increasingly early risks selecting hens that lay their eggs before the lights are turned on. Little is known about such behavior, which could decrease egg quality and increase off-nest laying.

The low to moderate heritability of NAL suggests that it has a genetic background. The objective of the NAL trait is to determine whether hens prefer to lay in the nests or not. However, NAL also depends on hens’ physiological ability to lay eggs or not, and the data recording system cannot differentiate the two phenomena. We assume that NAL is a combination of two phenomena that influence egg production negatively in cage-free systems (i.e. off-nest laying and physiological inability to lay), and selection against these phenomena could help improve egg production in cage-free systems.

In conclusion, the cage-housing system will be forbidden in the European Union in a few years. Electronic nests provide the advantage of recording individual nesting behavior daily in cage-free systems and seem to be a good way to decrease the use of individual cages for selection. Phenotypic and genetic variabilities observed in this study show that laying performances could be improved in these group-housing systems by using information about oviposition time in the nests. In this study, CN, MOT and NAL could be the most interesting traits to include in breeding programs in the aim to increase LRN and reduce off-nest laying. The middle of production seems also to be the best period to improve these nest-related traits. Future studies that include genomics and more animals would increase the accuracy of results. Genetic relationships between these traits and egg-quality traits also need to be studied to be able to include these traits in breeding programs. Finally, the genetic gain expressed by commercial crossbreed hens should be studied, as should possible genotype-by-environment interactions between the nucleus farm, using individual electronic nests, and commercial farms, using collective nests.

## Supporting information

S1 Table Phenotypic data statistics for the mean time of entry for nest visits with oviposition. (DOCX)

S2 Table Minimum (min) and maximum (max) value of mean oviposition time (hh:mm) for a given laying pattern in the nests at the peak and middle production. (DOCX)

S1 Appendix Clutch number calculation with data of individual electronic nests. (DOCX)
