# Supplementary material for: Nest acceptance, clutch, and oviposition traits are promising selection criteria to improve egg production in cage-free system
Source: PLoS One. 2021 May 20;16(5):e0251037. doi: 10.1371/journal.pone.0251037 (PMC8136716; doi:10.1371/journal.pone.0251037)
Supplement: S1 Table — (DOCX) [file pone.0251037.s001.docx]

**S1 Table. Phenotypic data statistics for the mean time of entry for nest visits with oviposition.**

|  | Rhode Island Red | | | | | White Leghorn | | | | |
| --- | --- | --- | --- | --- | --- | --- | --- | --- | --- | --- |
| Trait | n^a^ | Mean | SD^b^ | Min^c^ | Max^d^ | n^a^ | Mean | SD^b^ | Min^c^ | Max^d^ |
| MET1^e^ (hh:mm) | 848 | 01:38 | 01:07 | -00:20^f^ | 06:44 | 928 | 03:17 | 01:18 | -00:01^f^ | 06:37 |
| MET2^e^ (hh:mm) | 1,121 | 02:31 | 01:25 | -00:16^f^ | 06:35 | 886 | 03:04 | 01:00 | 00:17 | 06:59 |

^a^Number of hens per trait; ^b^Standard deviation; ^c^Minimum value; ^d^Maximum value

^e^MET, mean time of entry for nest visits with oviposition. The number after abbreviations indicates the breeding period: 1 for the peak (24-43 wks. of age) and 2 for the middle (44-64 wks. of age) of production.

^f^MET before the lights were turned on.
