# Supplementary material for: Nest acceptance, clutch, and oviposition traits are promising selection criteria to improve egg production in cage-free system
Source: PLoS One. 2021 May 20;16(5):e0251037. doi: 10.1371/journal.pone.0251037 (PMC8136716; doi:10.1371/journal.pone.0251037)
Supplement: S2 Table — (DOCX) [file pone.0251037.s002.docx]

**S2 Table. Minimum (Min) and maximum (Max) value of mean oviposition time (hh:mm) for a given laying pattern in the nests at the peak and middle production.**

|  | Rhode Island Red | | White Leghorn | |
| --- | --- | --- | --- | --- |
| Laying pattern in the nests | Min | Max | Min | Max |
| A (> 4 clutches)^a^ | -00:01^c^ | 05:26 | 00:44 | 07:10 |
| A (> 4 clutches)^b^ | 01:33 | 06:23 | 00:27 | 06:29 |
| B (2-4 clutches)^a^ | -00:01^c^ | 05:05 | 00:28 | 06:41 |
| B (2-4 clutches)^b^ | 00:13 | 05:56 | 01:13 | 05:29 |
| C (1 clutch)^a^ | -00:07^c^ | 04:33 | 01:16 | 05:21 |
| C (1 clutch)^b^ | 00:11 | 05:43 | 00:32 | 05:03 |

^a^Peak production (24-43 wks. of age); ^b^Middle of production (44-64 wks. of age)

^c^Mean oviposition time before the lights were turned on.
