# Supplementary material for: Nest acceptance, clutch, and oviposition traits are promising selection criteria to improve egg production in cage-free system
Source: PLoS One. 2021 May 20;16(5):e0251037. doi: 10.1371/journal.pone.0251037 (PMC8136716; doi:10.1371/journal.pone.0251037)
Supplement: S1 Appendix — (DOCX) [file pone.0251037.s003.docx]

**S1 Appendix. Clutch number calculation with data of individual electronic nests.**

**
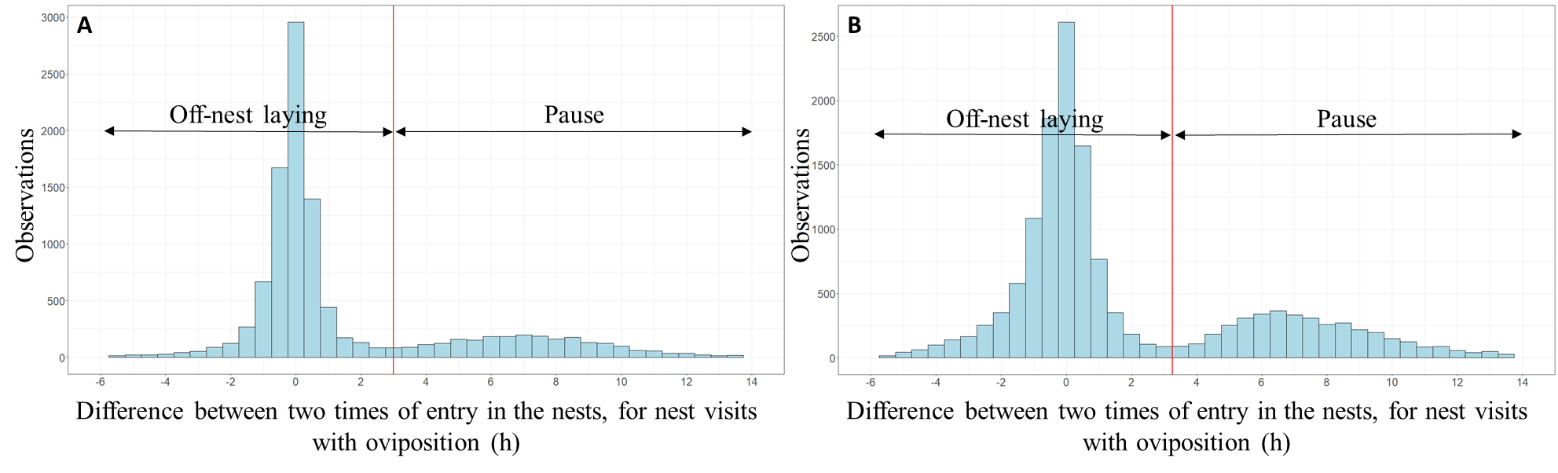
**A clutch is composed of eggs laid on consecutive days without a pause day. The first egg is laid early in the morning of the first day, and the last egg is laid later in the day of the last day. In the data of individual electronic nests, a day without laying in the nests could be a pause day or an off-nest laying day. When a hen did not lay in a nest for more than four days, its activity (i.e. pause or off-nest laying) was difficult to determine. When a hen did not lay in a nest for one to four days, however, we differentiated a pause from off-nest laying by observing the distribution of the difference between two times of entry in the nests, for nest visits with oviposition that flanked the days without laying in the nests (Fig A). A pause was defined as a period of one to four days where this time difference exceeded 3 h or 3 h 15 min for RIR and WL, respectively. If the time difference was shorter, we considered that the hen laid a number of off-nest eggs equal to the number of days without laying in the nests (i.e. up to 4 eggs). CN was equal to the number of pauses + 1. Hens with LRN ≥ 50% were used to analyze CN because only 0.1% and 0.3% of all periods without laying in the nests lasted more than four days for RIR and WL, respectively. For these hens, the off-nest laying rate could be calculated, but this trait was not analyzed because it had low phenotypic variance.

**Fig A. Histogram of the time difference between two times of entry in the nests, for nest visits with oviposition that flanked one to four days without laying in the nests for Rhode Island Red (A) and White Leghorn (B) lines.** Red lines represent thresholds used to differentiate a pause from an egg laid off-nest.
